# Supplementary material for: Activating lattice oxygen in high-entropy LDH for robust and durable water oxidation
Source: Nat Commun. 2023 Sep 27;14:6019. doi: 10.1038/s41467-023-41706-8 (PMC10533845; doi:10.1038/s41467-023-41706-8)
Supplement: Supplementary file 1 — Supplementary Information [file 41467_2023_41706_MOESM1_ESM.pdf]

Activating Lattice Oxygen in High-Entropy LDH for Robust and Durable Water Oxidation

Fangqing Wang[1, 2], Peichao Zou[3], Yangyang Zhang[2], Wenli Pan[4], Ying Li[1, 2], Limin Liang[2], Cong Chen[2], Hui Liu[1, 2*] and Shijian Zheng[1, 2*]

[1] Key Laboratory of Special Functional Materials for Ecological Environment and Information (Ministry of Education), Hebei University of Technology, Tianjin 300130, P. R. China

[2] School of Material Science and Engineering, Hebei University of Technology, Tianjin 300130, P. R. China

[3] Department of Physics and Astronomy, University of California, Irvine, CA 92697, USA

[4] Graduate School of Human and Environmental Studies, Kyoto University, Yoshida-nihonmatsu-cho, Sakyo, Kyoto 606-8501, Japan

E-mail: liuhui2013@hebut.edu.cn (Hui Liu); sjzheng@hebut.edu.cn (Shijian Zheng)

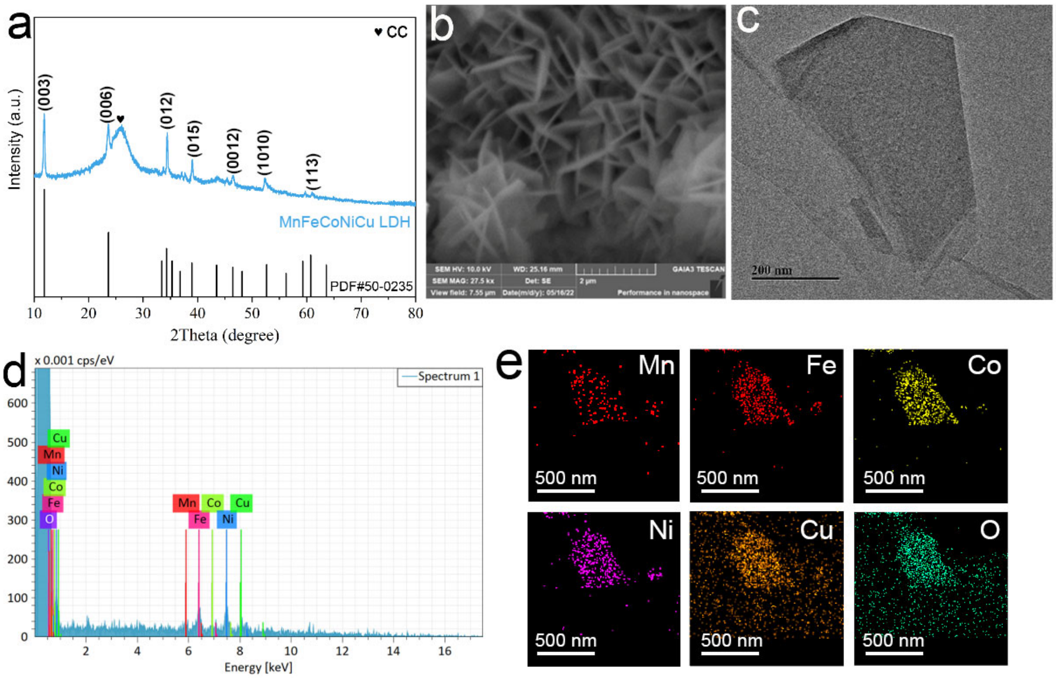

Figure S1. (a) XRD pattens, (b) SEM image, (c) TEM image, (d) EDS spectrum and (e) mapping of MnFeCoNiCu LDH. MnFeCoNiCu LDH is a single phase and all elements are uniformly distributed.

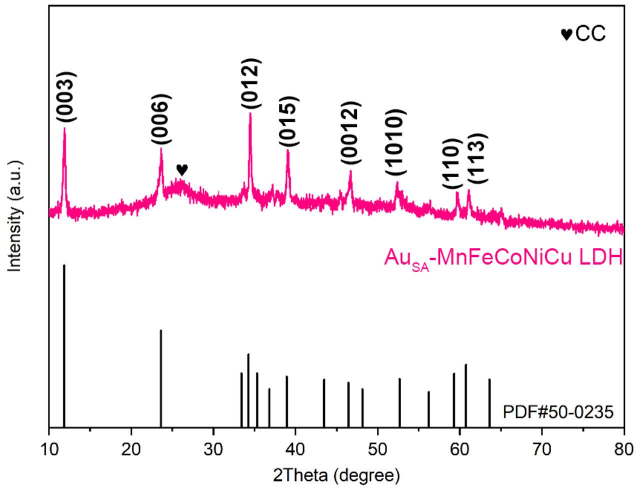

Figure S2. XRD pattens of Au_{SA}-MnFeCoNiCu LDH. Au_{SA}-MnFeCoNiCu LDH is a single phase.

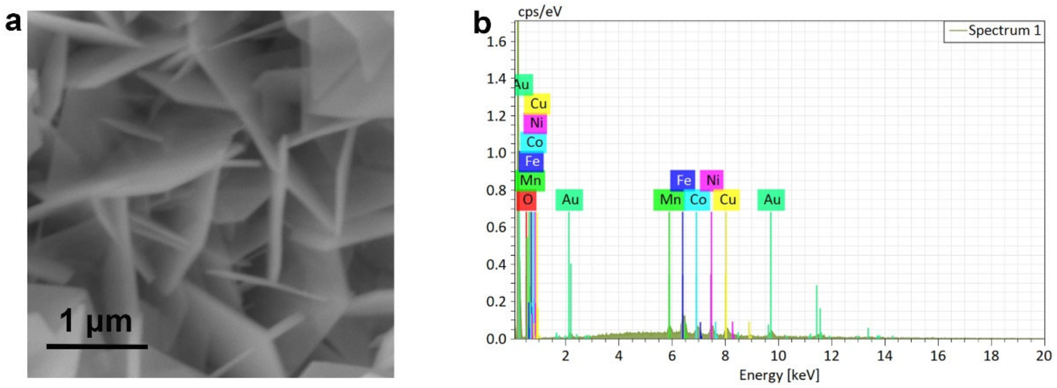

Figure S3. (a) SEM image and (b) EDS spectrum of Au_{SA}-MnFeCoNiCu LDH. Au_{SA}-MnFeCoNiCu LDH is edge-sharp nanosheets morphology.

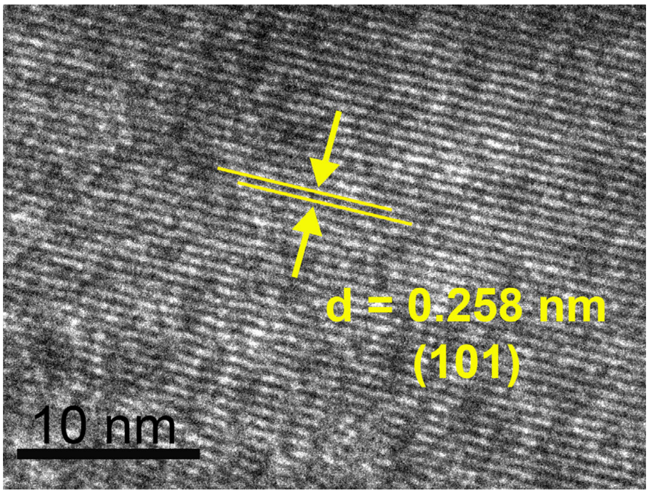

Figure S4. HRTEM image of MnFeCoNiCu LDH. 0.258 nm crystal spacing conforms to (101) crystal plane of MnFeCoNiCu LDH.

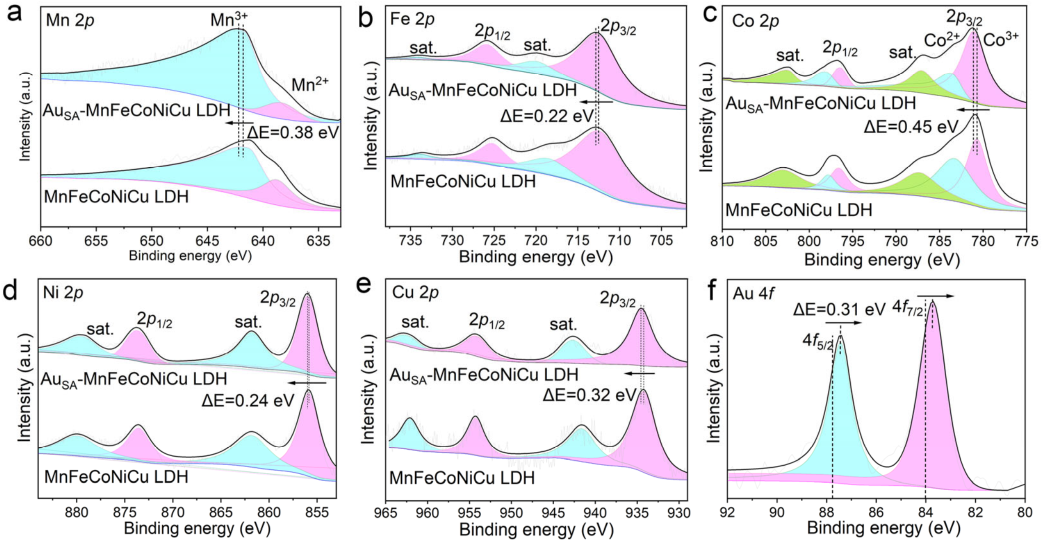

Figure S5. High-resolution XPS spectra of (a) Mn 2p, (b) Fe 2p, (c) Co 2p, (d) Ni 2p, (e) Cu 2p of Au_{SA}-MnFeCoNiCu LDH and MnFeCoNiCu LDH. (f) Au 4f of Au_{SA}-MnFeCoNiCu LDH.

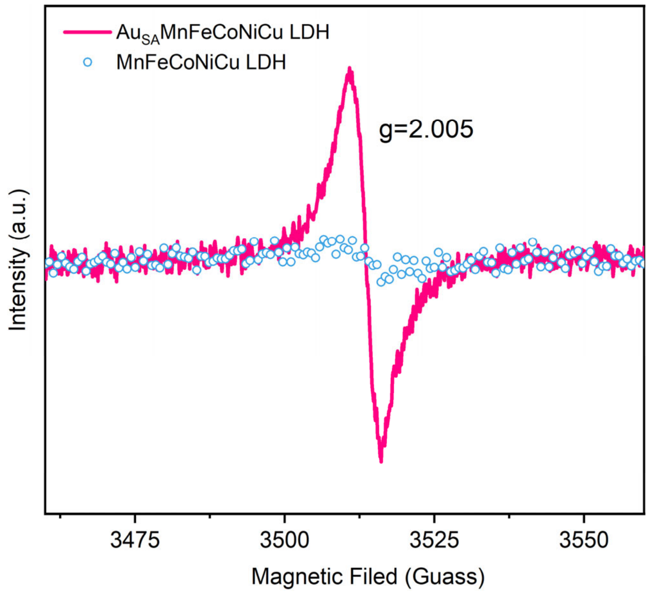

Figure S6. EPR spectra of Au_{SA}-MnFeCoNiCu LDH and MnFeCoNiCu LDH. Signal at g = 2.005 proves the presence of oxygen vacancies in Au_{SA}-MnFeCoNiCu LDH.

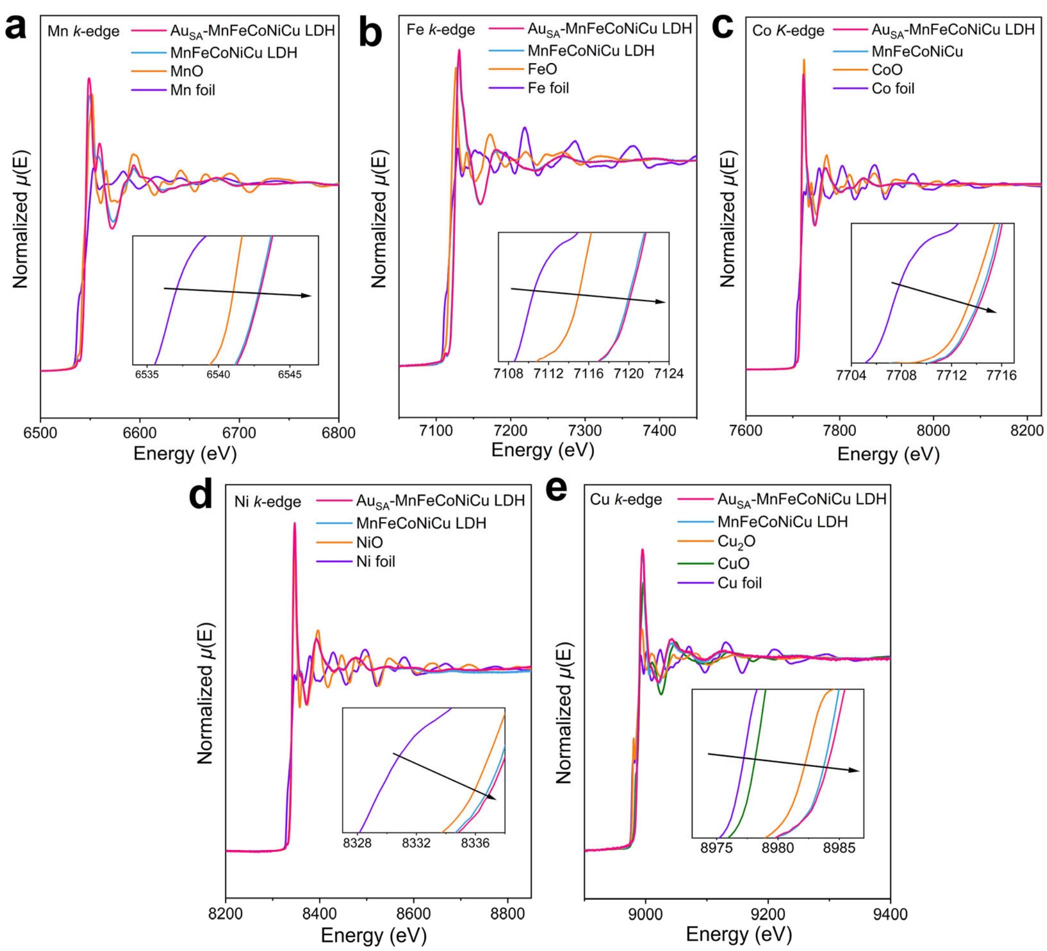

Figure S7. Normalized *K*-edge XAFS spectra of Mn, Fe, Co, Ni and Cu elements. Compared with those in MnFeCoNiCu LDH, all the absorption edges of Mn, Fe, Co, Ni and Cu in Au_{SA}-MnFeCoNiCu LDH shift to higher energies, implying an increased valence of 3d transition metals.

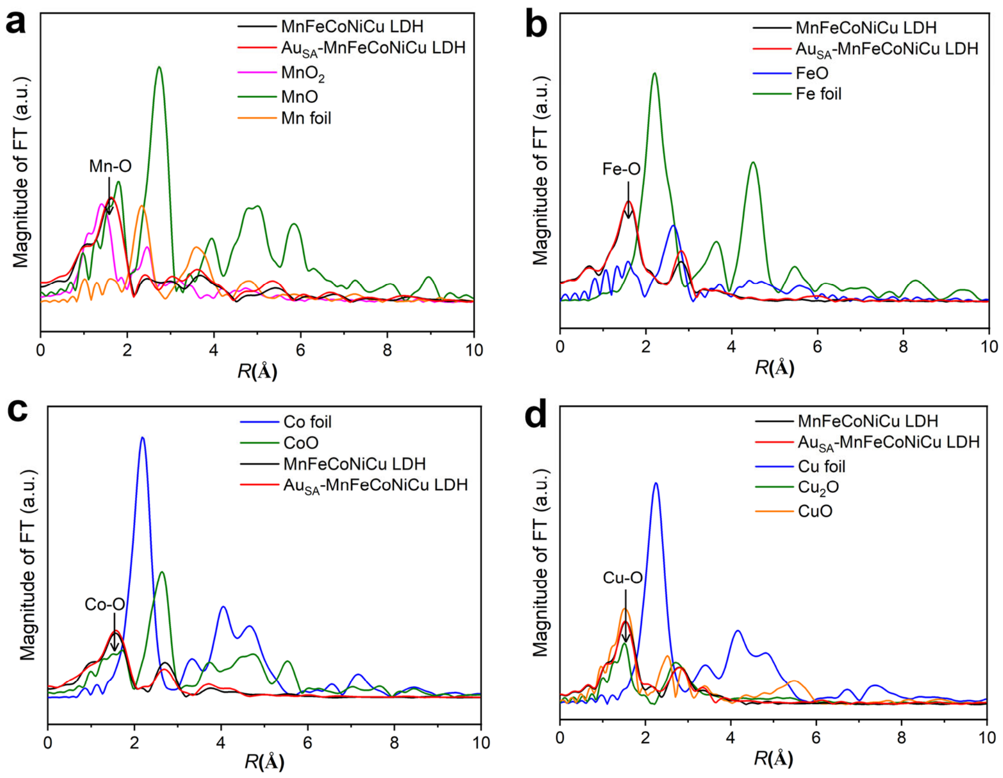

Figure S8. FT-EXAFS spectra and fitting results of Mn, Fe, Co and Cu elements. The changes in Mn-O, Fe-O, Co-O and Cu-O of Au_{SA}-MnFeCoNiCu LDH and MnFeCoNiCu LDH are almost negligible.

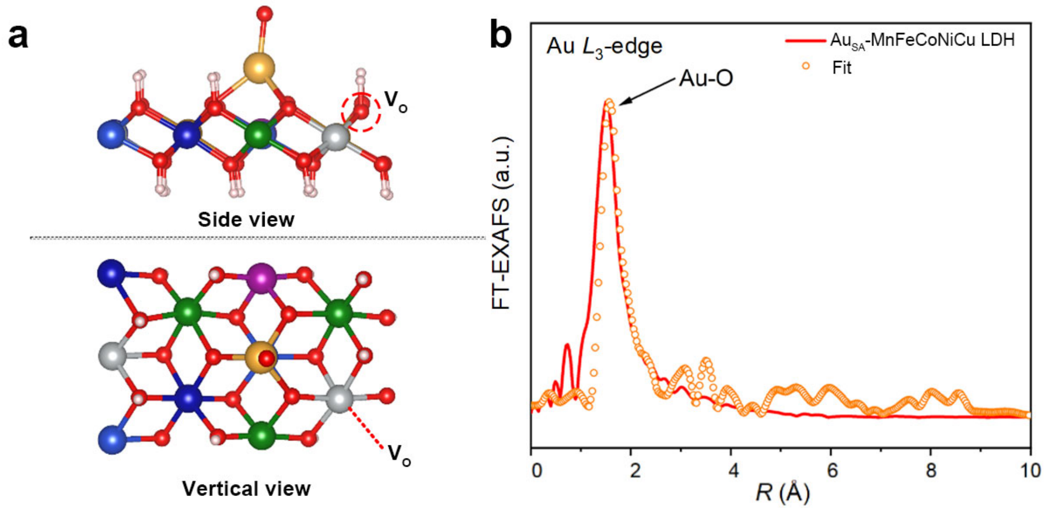

Figure S9. (a) The model in which Au is directly anchored by surface oxygen. (b) The corresponding EXAFS k^2 $\chi(k)$ Fourier transform (FT) spectra of Au_{SA}-MnFeCoNiCu LDH and fitting curve (Gray: Ni, Green: Fe, Navy: Co, Purple: Mn, Light Blue: Cu; Yellow: Au; Red: O; White: H).

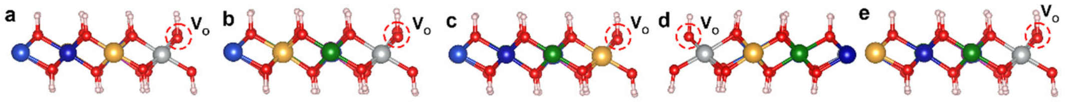

Figure S10. Model of Au Atom Replacing Surface (a) Fe, (b) Co, (c) Ni, (d) Mn, (e) Cu Sites (Gray: Ni, Green: Fe, Navy: Co, Purple: Mn, Light Blue: Cu; Yellow: Au; Red: O; White: H).

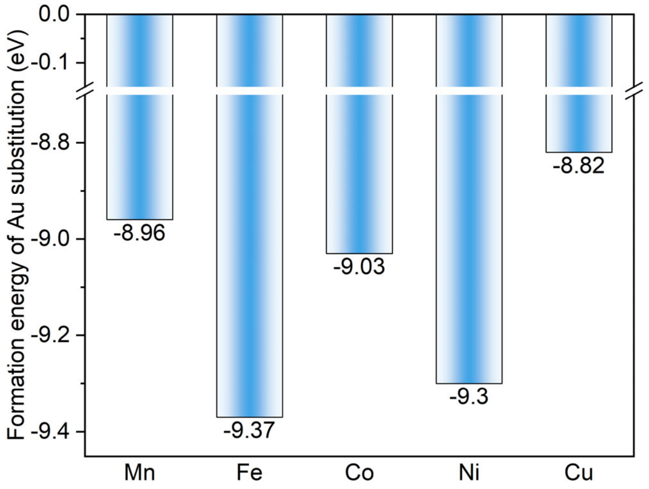

Figure S11. Calculated the substitution formation energies of Au_M (M=Fe, Co, Ni, Mn and Cu) via DFT. Au is easier to replace Fe (-9.37 eV) than Mn (-8.96 eV), Co (-9.03 eV), Ni (-9.30 eV) and Cu (-8.82 eV).

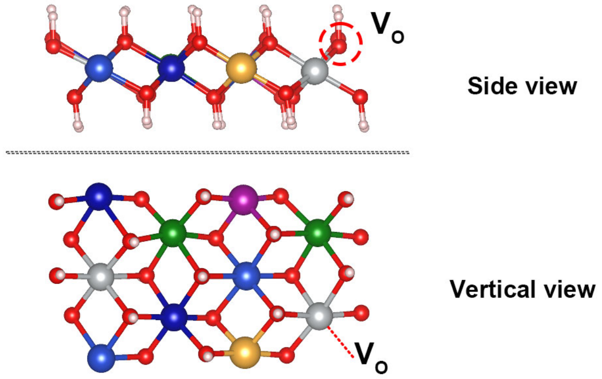

Figure S12. Model of Au occupying Fe site (Gray: Ni, Green: Fe, Navy: Co, Purple: Mn, Light Blue: Cu; Yellow: Au; Red: O; White: H).

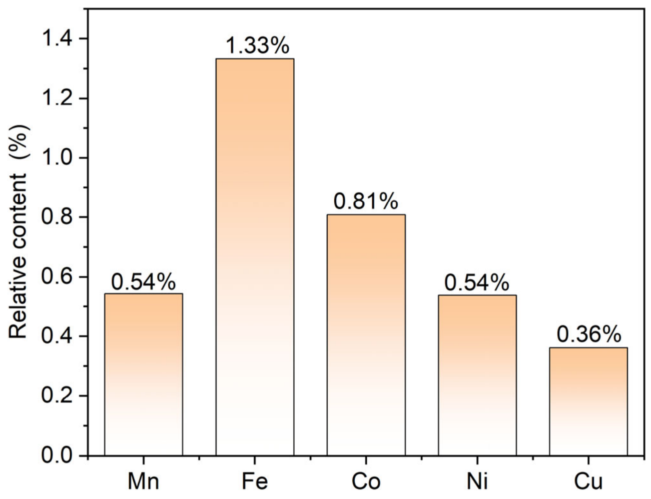

Figure S13. The proportion of metal elements dissolved in the electrolyte to the metal elements in the original sample during the CV process of MnFeCoNiCu LDH measured by ICP-MS, which stands for the metal dissolution percentage. Calculation method: the relative content is the ratio of the mass of a metal element (Mn, Fe, Co, Ni and Cu) dissolved in the electrolyte after CV to the mass of the element in the original sample, shown as formula below.

$$\text{Relative content} = \frac{C_d \times V}{C_o \times m}$$

Here C_d (mg L^{-1}) is the dissolved concentration of the element in the electrolyte after CV test, V (L) is the volume of electrolyte during CV test, C_0 (mg kg^{-1}) is the original loading content of the element on the electrode before CV test, and m (kg) is the mass of electrode.

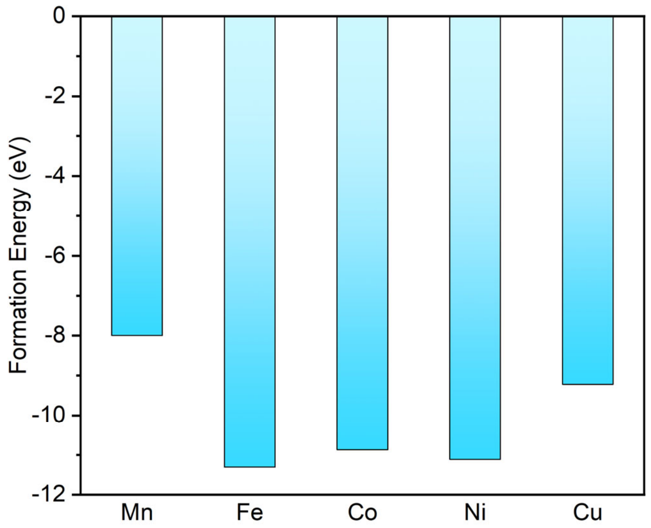

Figure S14. Metal-vacancy formation energy of Au_{SA}-MnFeCoNiCu LDH. From the

thermodynamic point of view, from a thermodynamic perspective, Fe vacancies are more likely to form.

Supplementary note 1. Investigate the effect of different iR corrections on the OER activity of Au_{SA}-MnFeCoNiCu LDH

To investigate the effect of different iR corrections on the OER activity of Au_{SA}-MnFeCoNiCu LDH, we carried out LSV tests at different iR compensations (95%, 80% and without compensation), and the results are shown in **Figure 3a** and **Figure S15**. Under 80% and 95% iR compensation, both of the LSV curves show nearly vertical lines at high current densities over 100 mA cm^{-2}. Even more the LSV curve without IR compensation exhibit a fast reaction kinetics at high current density, suggesting that the excellent activities are not attributed to the IR compensation but the distinguished intrinsic activity. Moreover, under different IR compensation conditions, the OER activity of Au_{SA}-MnFeCoNiCu LDH is higher than MnFeCoNiCu LDH.

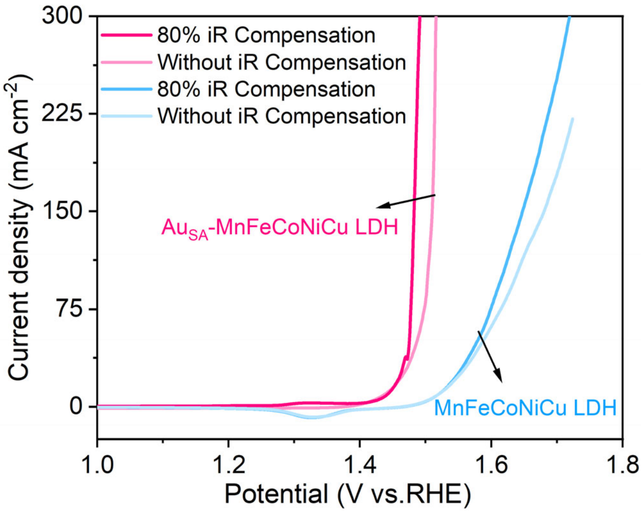

Figure S15. LSV curves (scan rate is 2 mV s^{-1}) of Au_{SA}-MnFeCoNiCu LDH (pink line) and MnFeCoNiCu LDH (blue line) with different IR corrections in 1.0 M KOH (pH=14). The loading of catalysts is ~1 mg cm^{-2}, and the solution resistance is 2.0 Ω.

Supplementary note 2. Comparison of the activity and stability of Au_{SA}-MnFeCoNiCu LDH and 20 other materials with different compositions of high entropy LDHs decorated with single-atom Au (Au_{SA}-HE LDHs).

We have synthesized 20 high-entropy LDHs materials decorated with single-atom Au with different compositions (Au_{SA}-HE LDHs) besides Au_{SA}-MnFeCoNiCu LDH. The XRD, EDS and elemental mapping image as shown in **Figure S16**, **Figure S17 and Table S3** confirm the successful formation of single high-entropy phase. The LSV curves of 20 high-entropy samples with different composition are provided in **Figure S18**. Moreover, the LSV curves after 10 000 CV cycles of the samples are also offered to reflect the stability (**Figure S19** and **Figure S20)**. It can be clearly seen that Au_{SA}-MnFeCoNiCu LDH exhibit the lowest overpotential at 10 mA cm^{-2} and the smallest positive shift after 10000 CV cycles among all the samples, implying the best OER activity and stability, which also indicate the optimal composition.

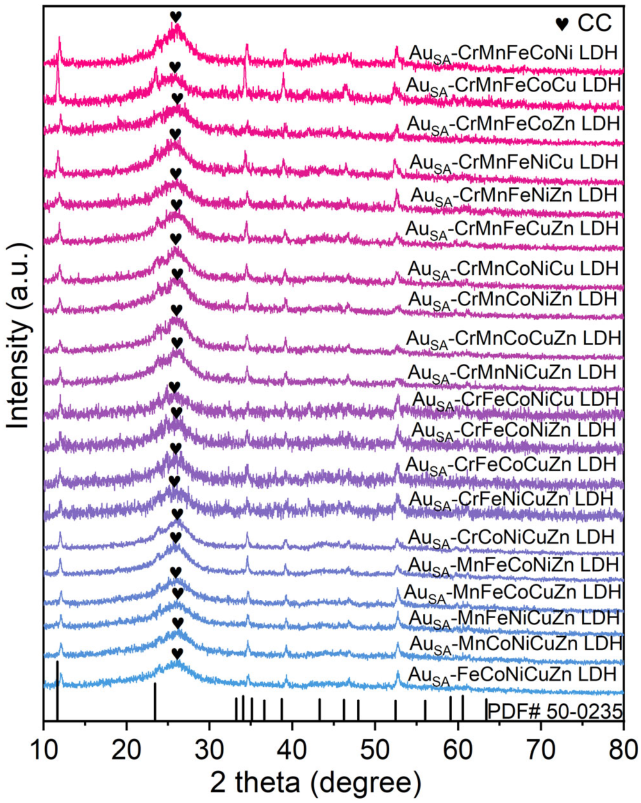

Figure S16. XRD spectra of 20 Au_{SA}-HE LDHs.

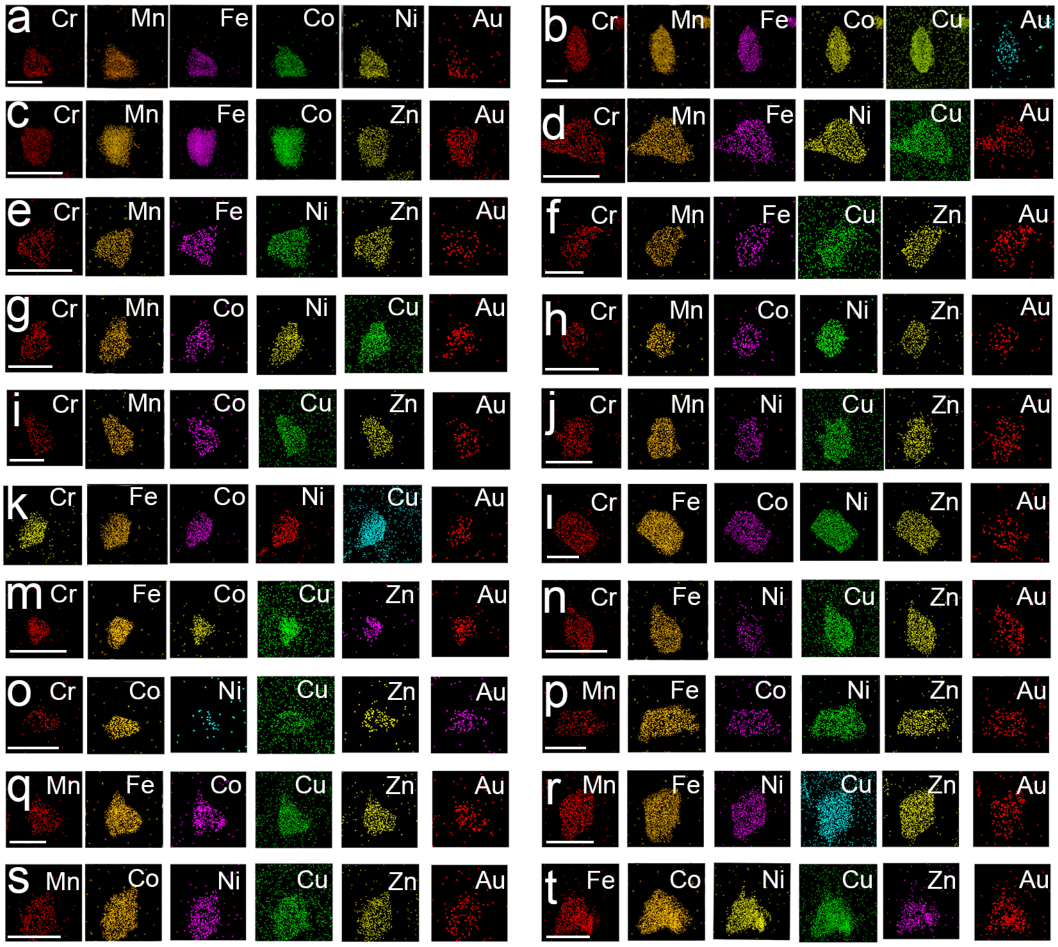

Figure S17. Elemental mapping of 20 Au_{SA}- HE LDHs, scale bar is 250 nm.

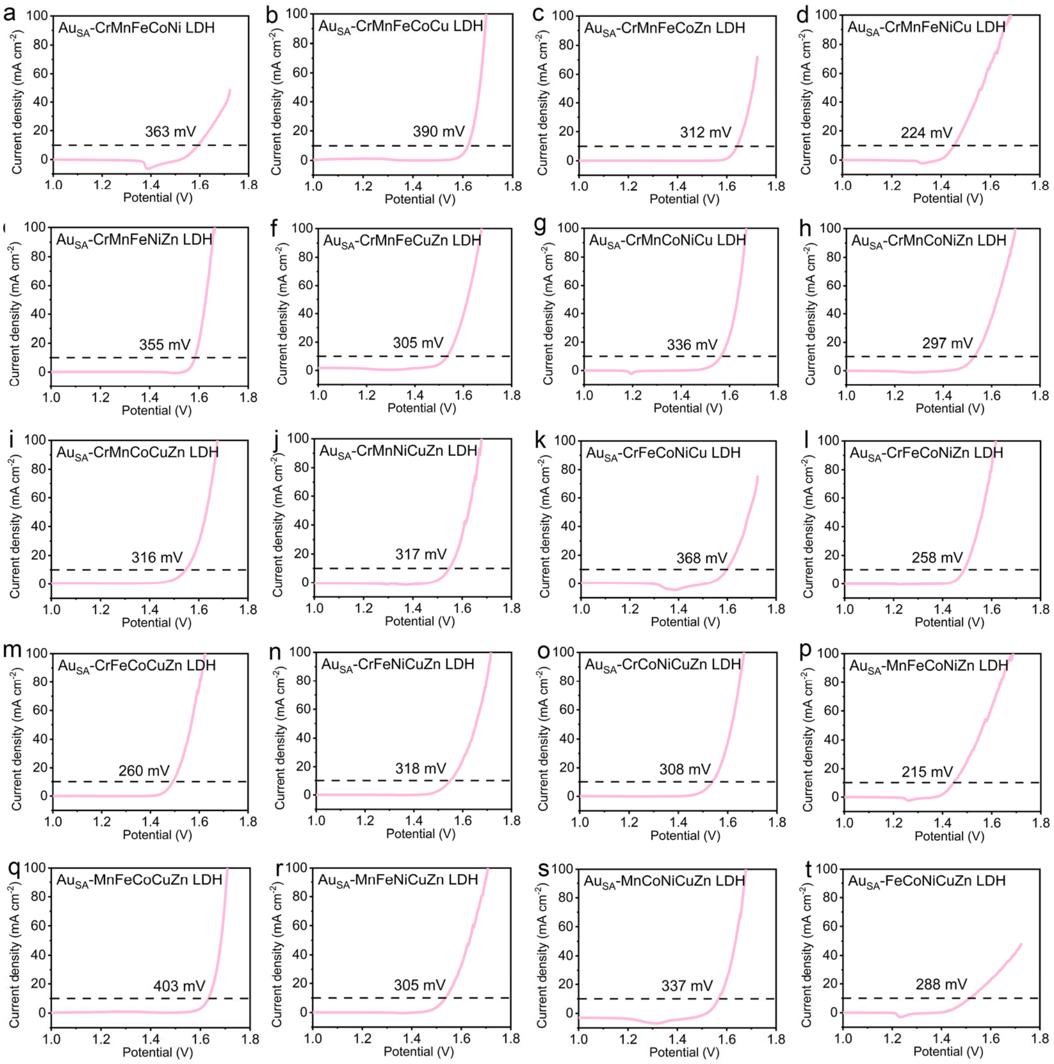

Figure S18. LSV curves (scan rate is 2 mV s^{-1}) of 20 Au$_{SA}$-HE LDHs in 1.0 M KOH (pH=14). The loading of all catalysts is ~1 mg cm^{-2}, and the solution resistance is 2.0 Ω.

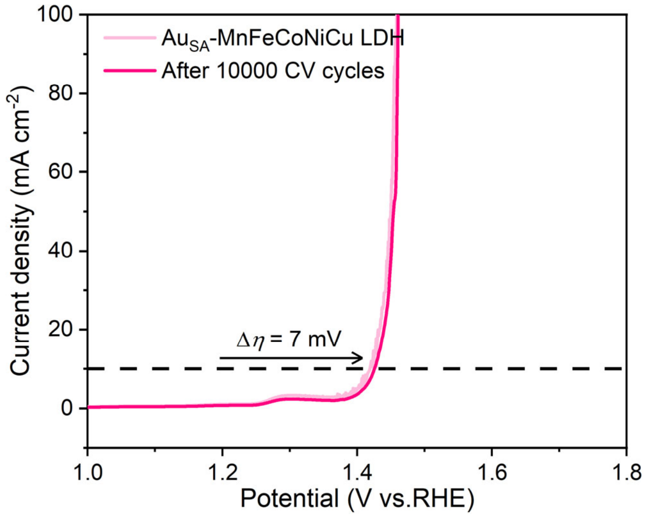

Figure S19. LSV curves (scan rate is 2 mV s^{-1}) before and after 10000 CV cycles of

Au$_{SA}$-MnFeCoNiCu LDH in 1.0 M KOH (pH=14). The load of catalyst is ~1 mg cm^{-2}, and the solution resistance is 2.0 Ω.

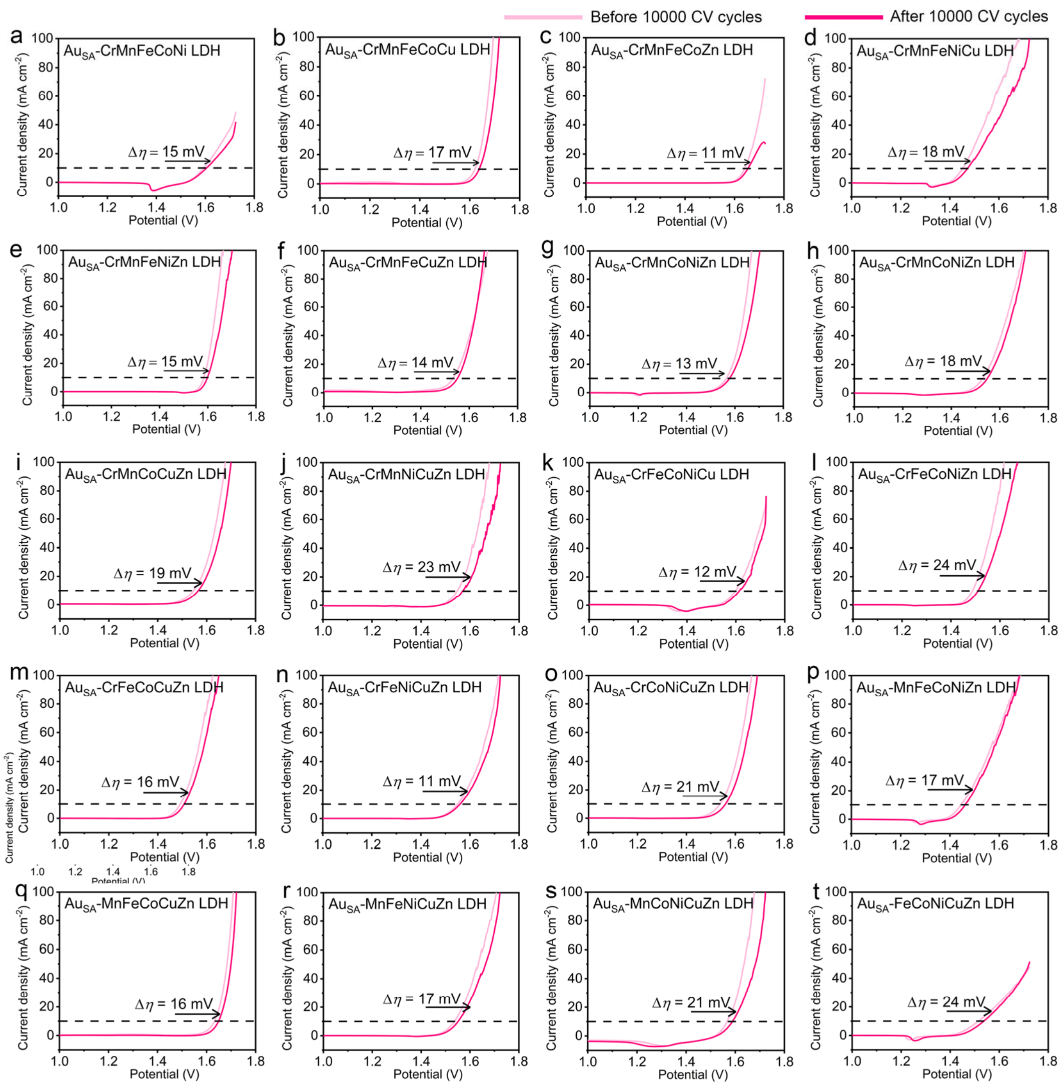

Figure S20. LSV shift of comparative 20 Au$_{SA}$-HE LDHs.

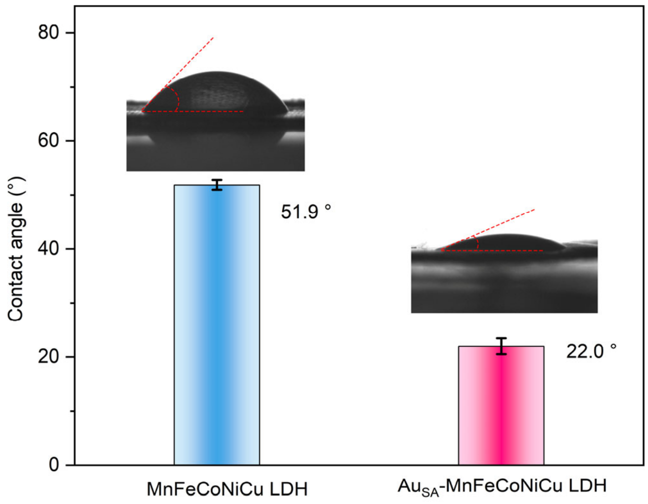

Figure S21. Contact angle of liquid drop on catalyst surface.

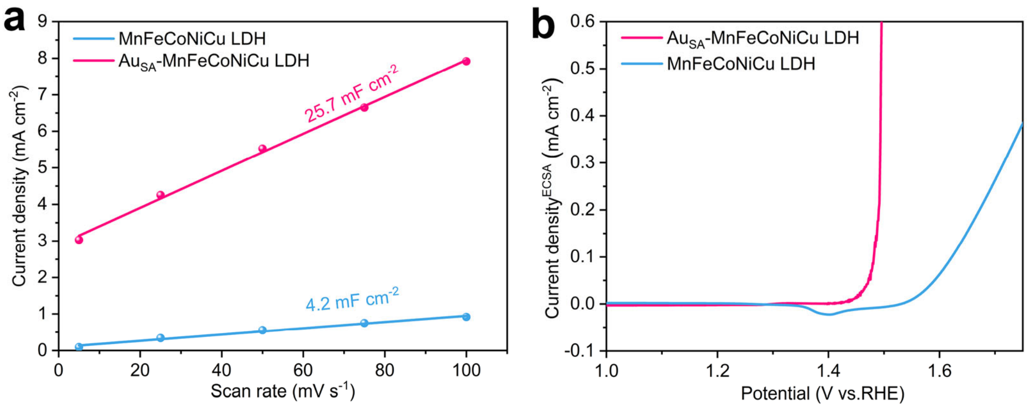

Figure S22. (a) C_{dl} curves, (b) LSV curves normalized by ECSA of the Au_{SA}-MnFeCoNiCu LDH and MnFeCoNiCu LDH. Tests were performed in 1.0 M KOH (pH=14).

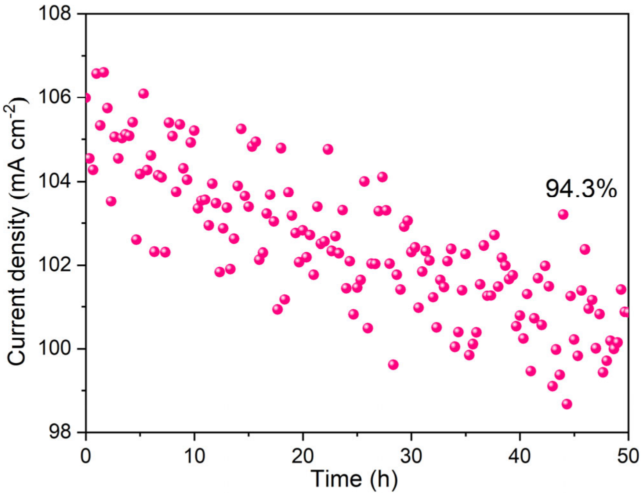

Figure S23. Stability test of Au_{SA}-MnFeCoNiCu LDH at the initial 50 h in 1.0 M KOH (pH=14), activity decay is 94.3 %.

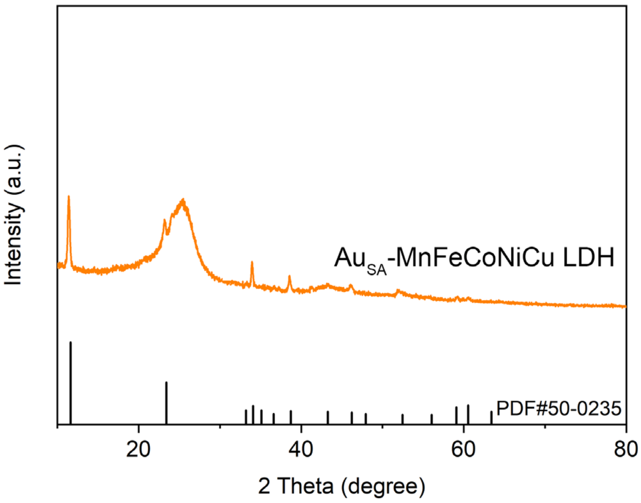

Figure S24. XRD pattens of Au_{SA}-MnFeCoNiCu LDH after 50 h Stability test.

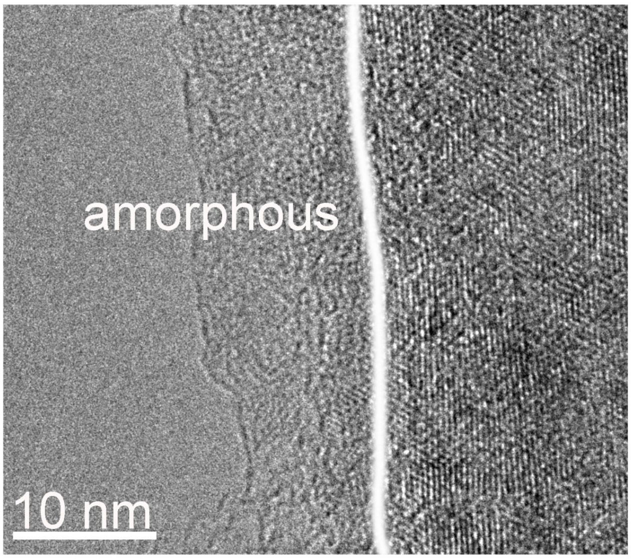

Figure S25. HR-TEM images of Au_{SA}-MnFeCoNiCu LDH after 50 h long-term OER test.

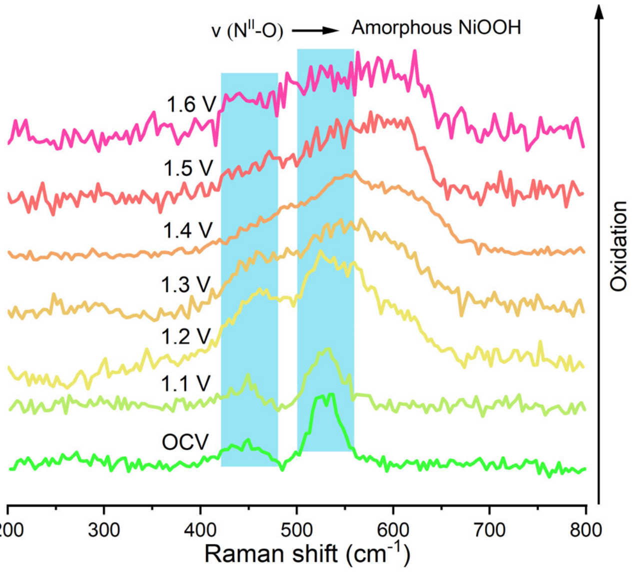

Figure S26. In situ Raman spectra of Au_{SA}-MnFeCoNiCu LDH. With the increase of the OER potential, two narrow peaks corresponding to the Ni^{II}-O bond gradually weaken until they disappear, while a broad peak gradually appears, which corresponds to the NiOOH (MOOH) species. Combined with the Mapping results of Figure S27, it shows that with the OER process, the Au_{SA}-MnFeCoNiCu LDH surface gradually transforms to amorphous Au_{SA}-MnFeCoNiCuOOH.

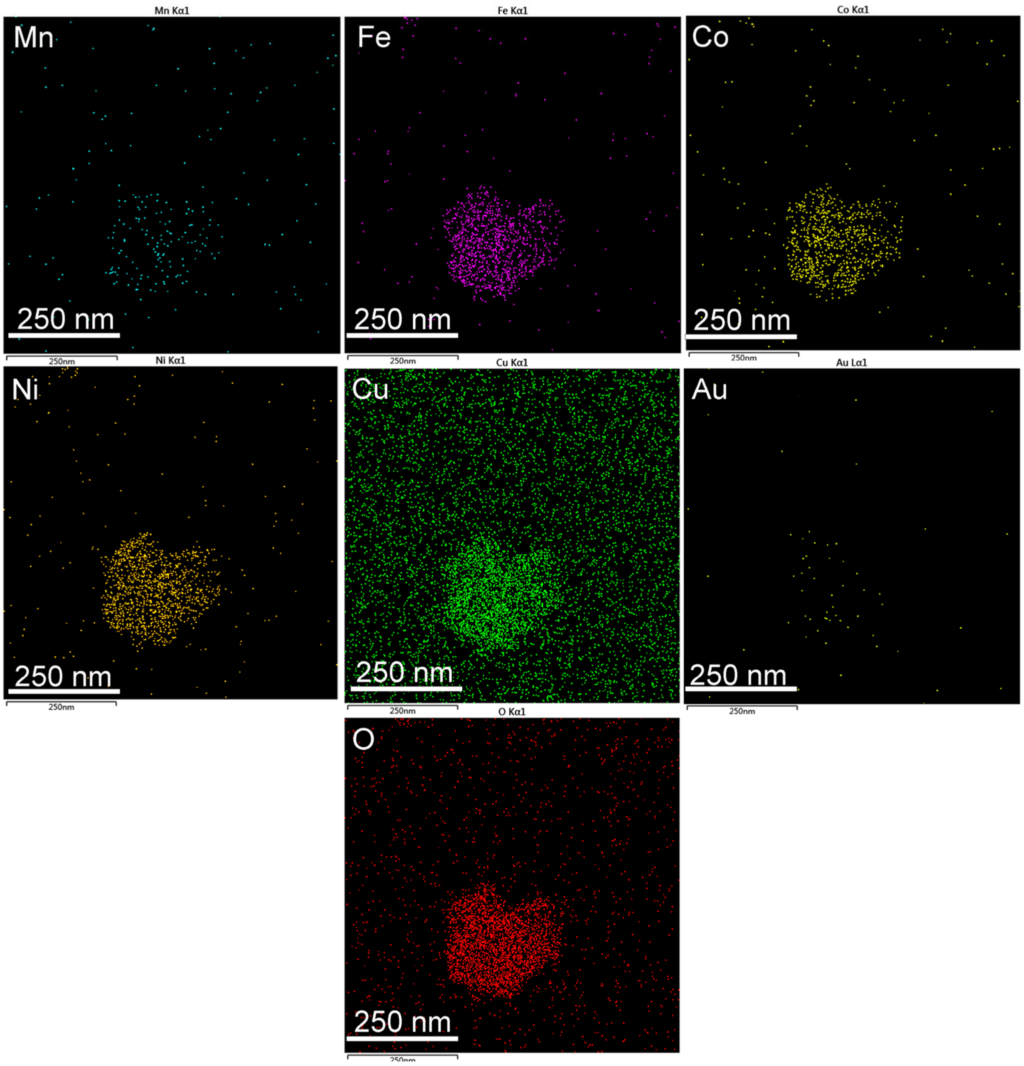

Figure S27. Element mapping of Au_{SA}-MnFeCoNiCu LDH after 50 h long-term OER test.

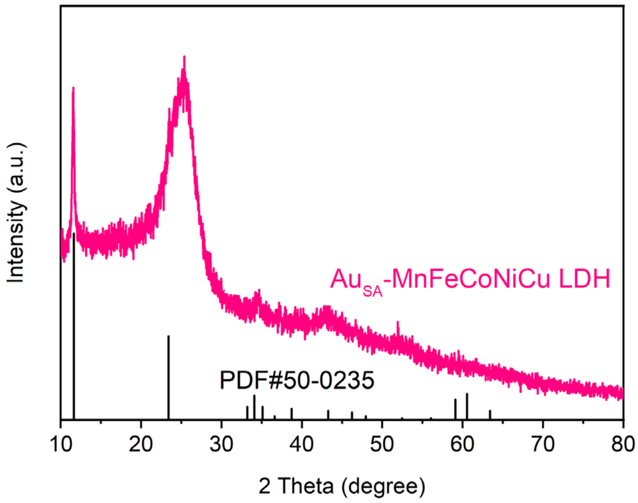

Figure S28. XRD pattens of Au_{SA}-MnFeCoNiCu LDH after 700 h long-term OER test.

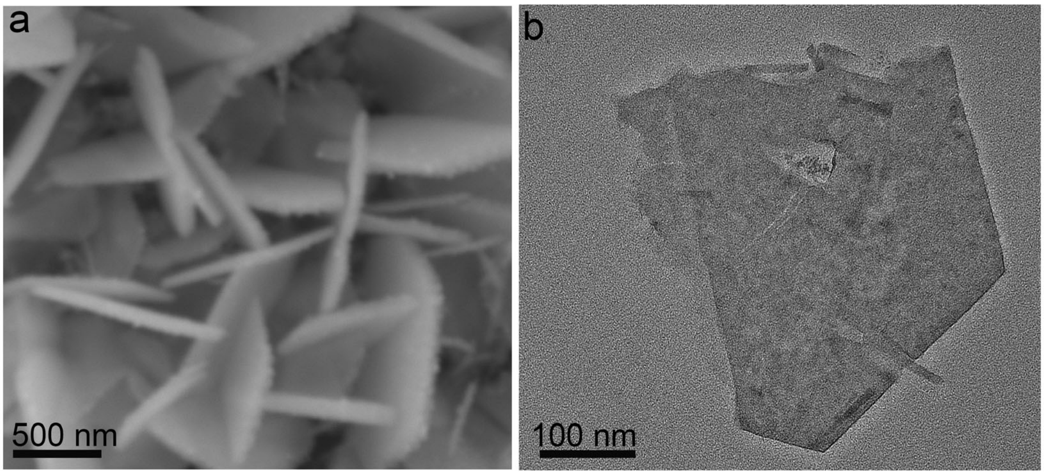

Figure S29. (a) SEM and (b) TEM images of Au_{SA}-MnFeCoNiCu LDH after 700 h long-term OER test.

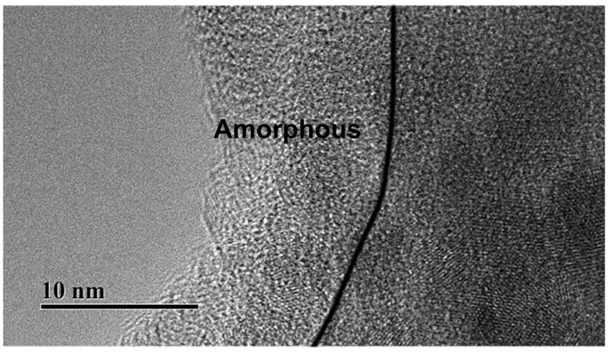

Figure S30. HRTEM image of Au_{SA}-MnFeCoNiCu LDH after 700 h long-term OER test.

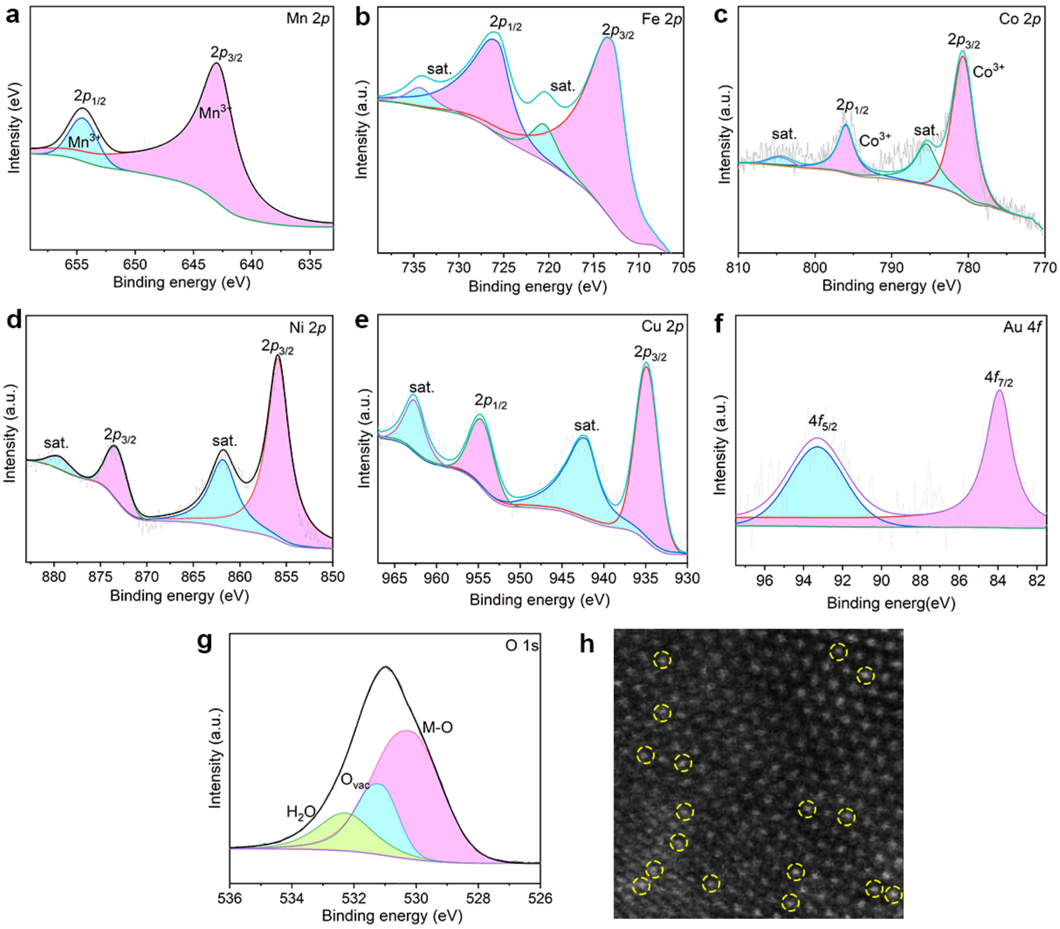

Figure S31. High-resolution XPS spectra of (a) Mn 2p, (b) Fe 2p, (c) Co 2p, (d) Ni 2p, (e) Cu 2p (f) Au 4f and (g) O 1s of Au_{SA}-MnFeCoNiCu LDH after stability test. (h) AC-HAADF-STEM image of Au_{SA}-MnFeCoNiCu LDH after 700 h long-term OER test.

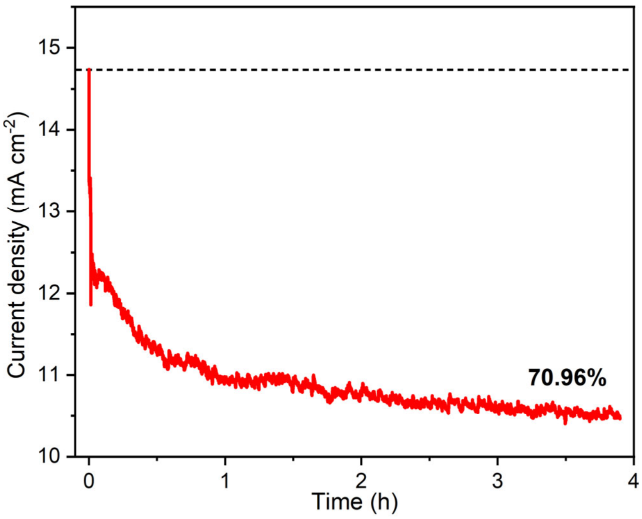

Figure S32. Stability test of NiFe LDH.

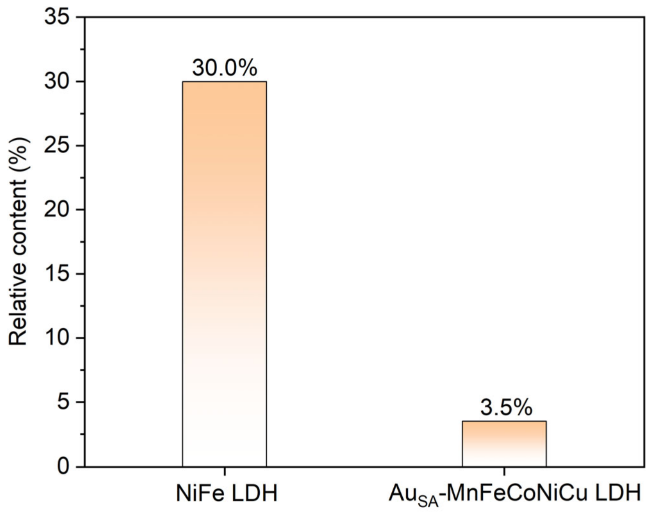

Figure S33. After the stability test of NiFe LDH and Au$_{SA}$-MnFeCoNiCu LDH, the proportion of Fe element dissolved in the electrolyte to the Fe element in the original sample, which stands for the Fe dissolution percentage. The calculation method is similar to Figure S13.

$$\text{Relative content} = \frac{C_d \times V}{C_o \times m}$$

Here C_d (mg L^{-1}) is the dissolved concentration of the Fe element in the electrolyte after stability test, V (L) is the volume of electrolyte during stability test, C_0 (mg kg^{-1}) is the original loading content of the Fe element on the electrode before stability test, and m (kg) is the mass of electrode.

Supplementary note 3. HER performance testing of Au$_{SA}$-MnFeCoNiCu LDH and MnFeCoNiCu LDH.

The HER properties of Au$_{SA}$-MnFeCoNiCu LDH and MnFeCoNiCu LDH were tested, as shown in **Figure S34**. The results show that the HER activity of Au$_{SA}$-MnFeCoNiCu LDH is not very good, which may be due to the poor electrical conductivity of the LDH material.

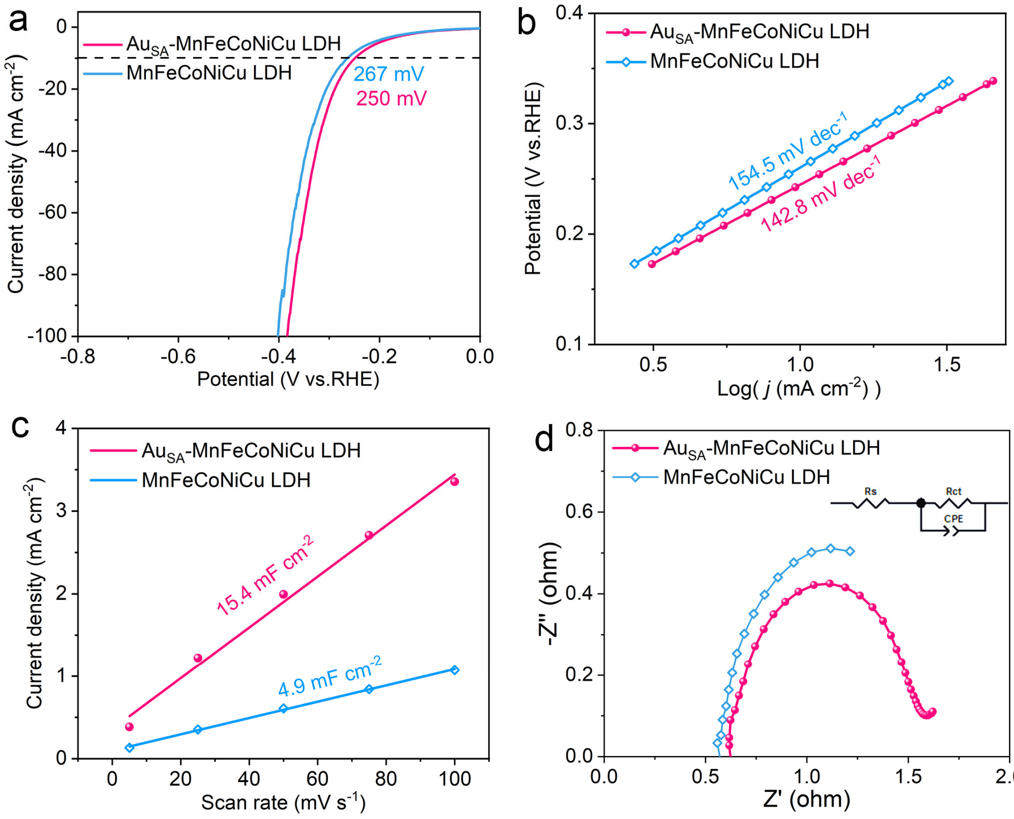

Figure S34. HER performance of Au_{SA}-MnFeCoNiCu LDH. (a) LSV curves (scan rate is 2 mV s^{-1}), (b)Tafel plots, (c) C_{dl} plots, and (d) EIS curves (The frequency range were set to $10^6 \sim 10^{-2}$ Hz). All tests are performed in the 1.0 M KOH (pH=14) solution, and the solution resistance is 1.8 Ω.

Supplementary note 4. Uncovering the relationship between the electronegativity of doped metal atoms and the mechanism of oxygen evolution.

To further reveal the relationship between the electronegativity of the incorporated metal atoms and the oxygen evolution mechanism, we choose other three metals with different electronegativity (Ru: 2.2, Pt: 2.3 and Ag: 1.9) for comparison. The structure characterization results validate the successful incorporation of single-atom Ru, Pt and Ag (**Figure S35-S37 and Table S6**). The LSV curves of different single-atom incorporation on MnFeCoNiCu LDH is displayed in **Figure S38**. Subsequently, we investigate the pH-dependent properties of high-entropy LDH catalysts incorporated with different single atoms, as shown in **Figure S39**, and the ρ^{RHE} value is utilized to

evaluate the pH-dependent properties. The results reveal that there is a positive correlation between the electronegativity and the pH-dependent properties, implying that incorporating the single-atom metal with higher electronegativity is more conductive to triggering LOM.

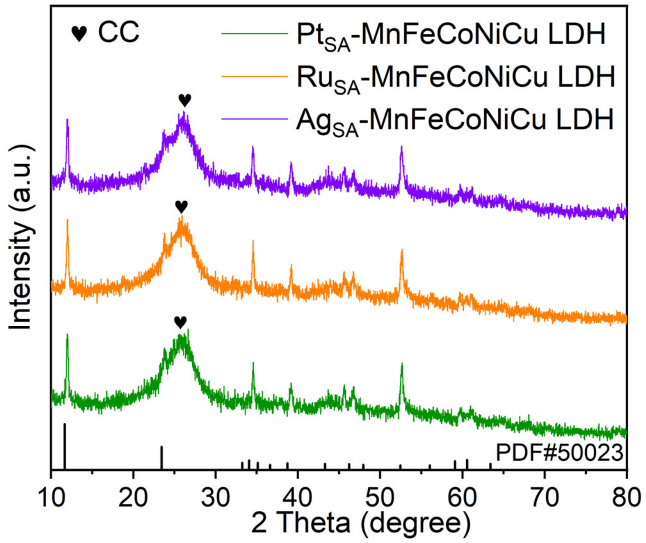

Figure S35. XRD pattens of different single-atom incorporation on MnFeCoNiCu LDH.

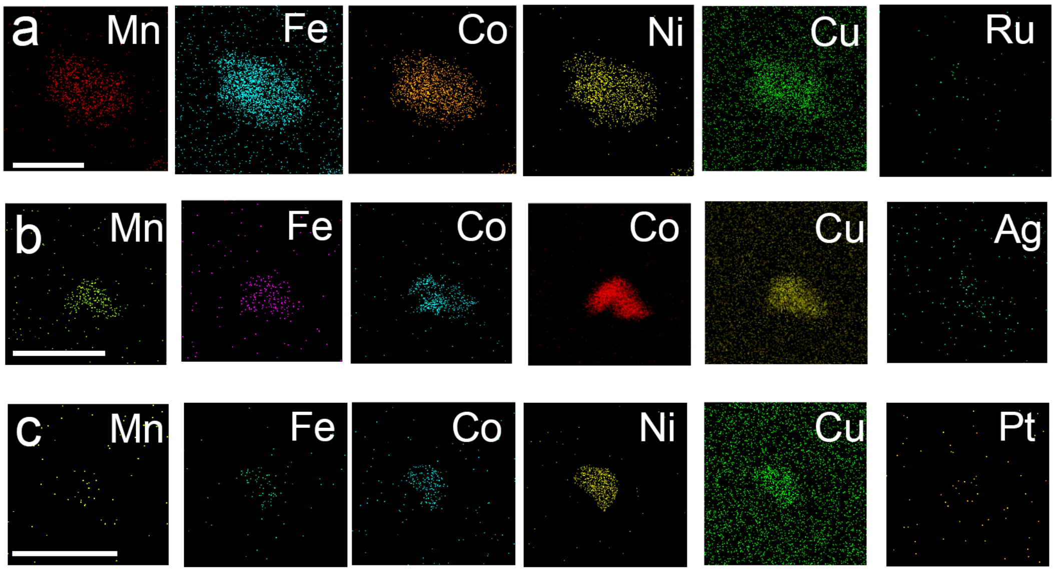

Figure S36. Element mapping of Ru_{SA}-MnFeCoNiCu LDH, Ag_{SA}-MnFeCoNiCu LDH and Pt_{SA}-MnFeCoNiCu LDH (Scale bar is 1 μm).

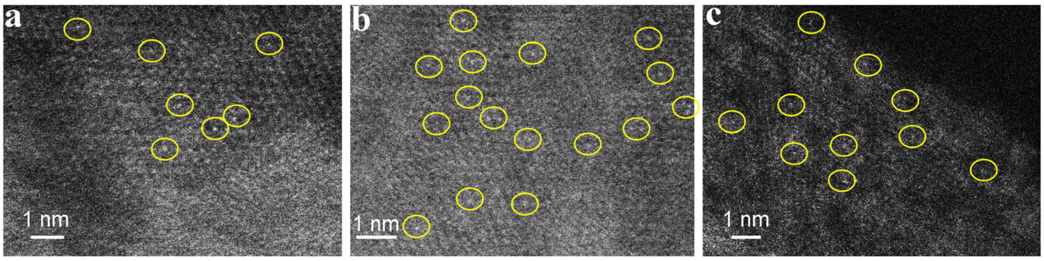

Figure S37. AC-HAADF-STEM images of (a) Pt_{SA}-MnFeCoNiCu LDH, (b) Ru_{SA}-MnFeCoNiCu LDH and (c) Ag_{SA}-MnFeCoNiCu LDH.

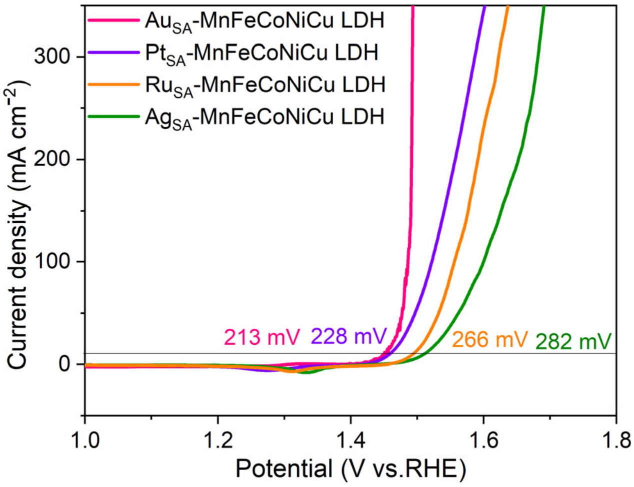

Figure S38. LSV curves (scan rate is 2 mV s^{-1}) of Au_{SA}-MnFeCoNiCu LDH, Pt_{SA}-MnFeCoNiCu LDH, Ru_{SA}-MnFeCoNiCu LDH and Ag_{SA}-MnFeCoNiCu LDH in 1.0 M KOH (pH=14). The loading of catalysts is ~1 mg cm^{-2}, and the solution resistance is 2.0 Ω.

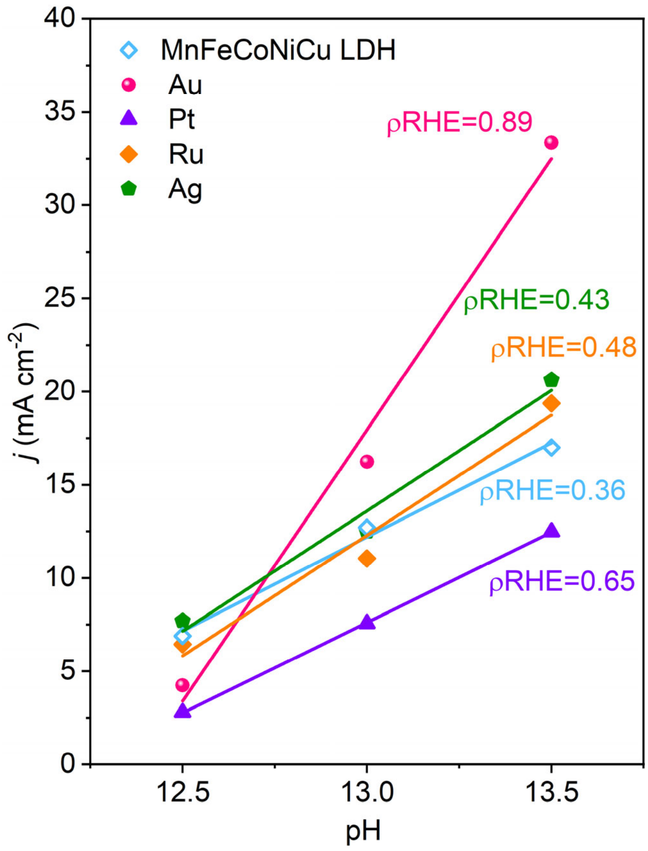

Figure S39. ρ^{RHE} of different single-atom incorporation at 1.45 V vs. RHE.

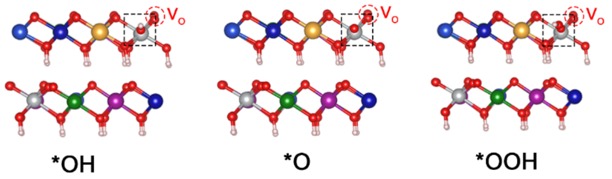

Figure S40. The theoretical models of AEM on Au_{SA}-MnFeCoNiCuOOH involved the adsorption of *OH, *O, *OOH (Ni is active site). (Gray: Ni, Green: Fe, Navy: Co, Purple: Mn, Light Blue: Cu; Yellow: Au; Red: O; White: H).

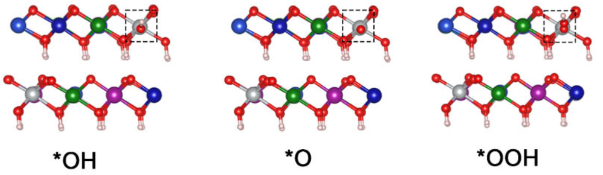

Figure S41. The theoretical models of AEM on MnFeCoNiCuOOH involved the adsorption of *OH, *O, *OOH (Ni is active site). (Gray: Ni, Green: Fe, Navy: Co, Purple: Mn, Light Blue: Cu; Red: O; White: H).

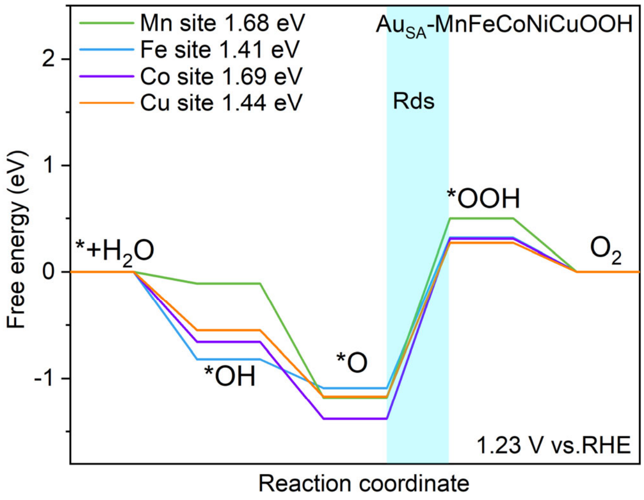

Figure S42. Free energy profiles (AEM) for Mn Fe, Co, Ni and Cu sites of Au$_{SA}$-MnFeCoNiCuOOH.

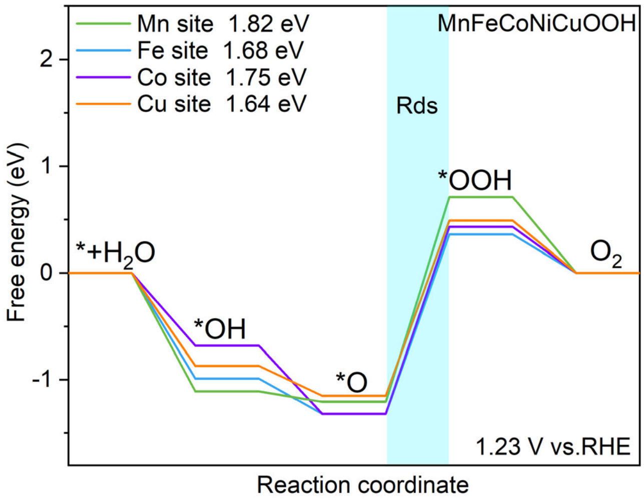

Figure S43. Free energy profiles (AEM) for Mn Fe, Co, Ni and Cu sites of MnFeCoNiCuOOH.

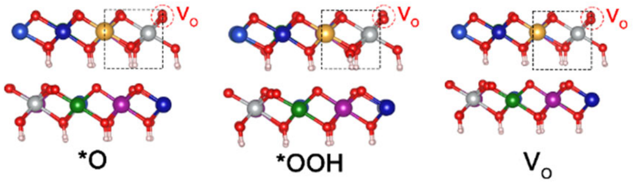

Figure S44. The theoretical models of LOM on Au$_{SA}$-MnFeCoNiCuOOH involved the adsorption of *O, *OOH, and Vo. (Gray: Ni, Green: Fe, Navy: Co, Purple: Mn, Light Blue: Cu; Yellow: Au; Red: O; White: H).

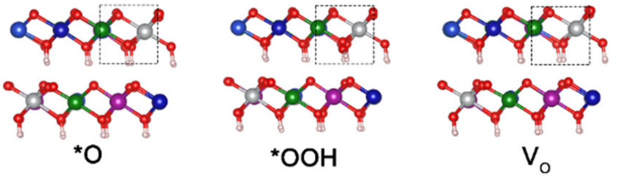

Figure S45. The theoretical models of LOM on MnFeCoNiCuOOH involved the adsorption of *O, *OOH, and Vo. (Gray: Ni, Green: Fe, Navy: Co, Purple: Mn, Light Blue: Cu; Red: O; White: H).

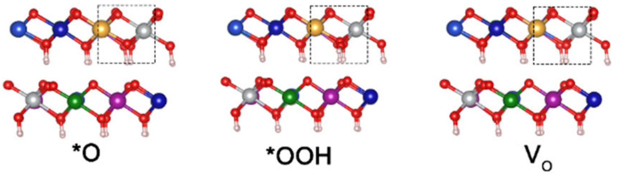

Figure S46. The theoretical models of LOM on MnFeCoNiCuOOH with only Au atoms involved the adsorption of *O, *OOH, and Vo. (Gray: Ni, Green: Fe, Navy: Co, Purple: Mn, Light Blue: Cu; Yellow: Au; Red: O; White: H).

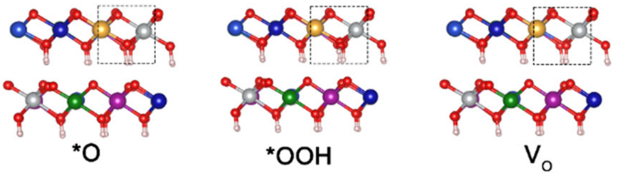

Figure S47. The theoretical models of LOM on MnFeCoNiCuOOH with only O vacancies involved the adsorption of *O, *OOH, and Vo. (Gray: Ni, Green: Fe, Navy: Co, Purple: Mn, Light Blue: Cu; Red: O; White: H).

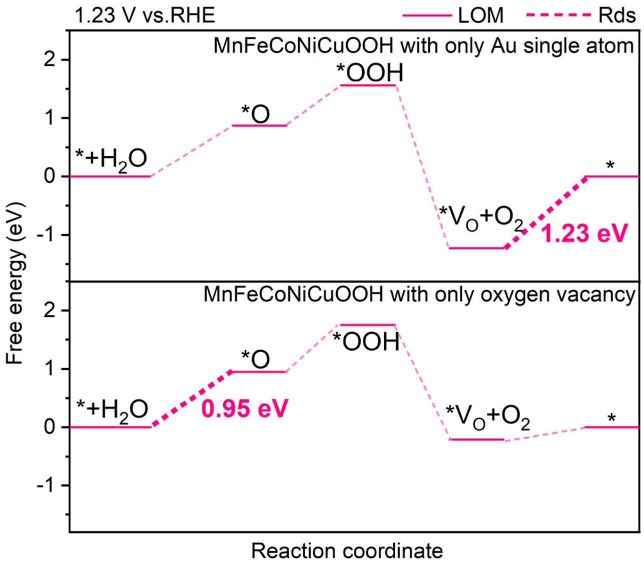

Figure S48. Free energies of OER via LOM on MnFeCoNiCuOOH with only Au atom and MnFeCoNiCuOOH with only O vacancy.

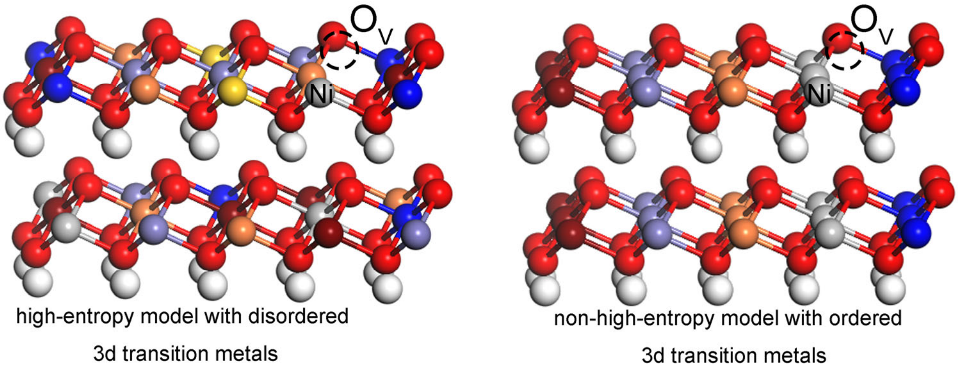

Figure S49. high-entropy Au_{SA}-MnFeCoNiCuOOH model with disordered 3d transition metals and non-high-entropy Au_{SA}-MnFeCoNiCuOOH model with ordered 3d transition metals (Gray: Ni, gold: Au, orange: Co, navy: Cu, purple: Fe, dark red: Mn, red: O, and white: H).

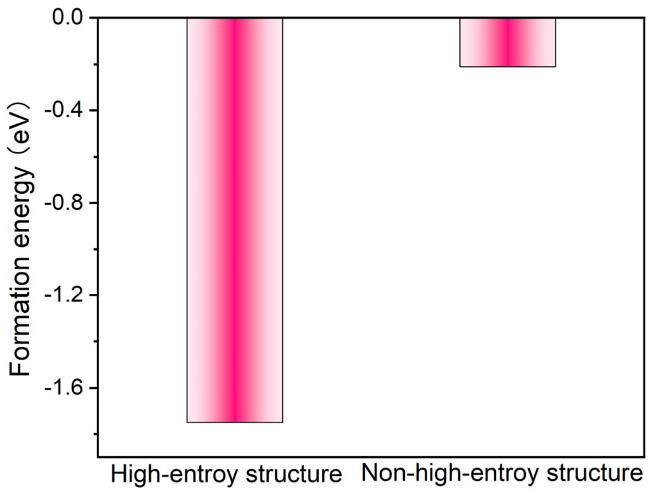

Figure S50. The formation energy of high-entropy structure and non-high-entropy structure.

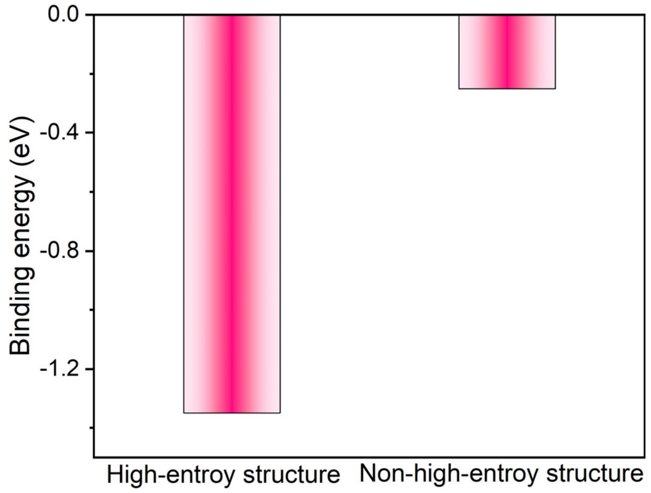

Figure S51. The binding energy of Ni next to O vacancy in high-entropy structure and non-high-entropy structure.

Table S1. EDS results of MnFeCoNiCu LDH and Au_{SA}-MnFeCoNiCu LDH.

	Mn (at%)	Fe (at%)	Co (at%)	Ni (at%)	Cu (at%)	Au (at%)	O (at%)
MnFeCoNiCu LDH	3.31	7.88	4.16	8.54	7.38	-	68.73

Au_{SA}-MnFeCoNiCu LDH	4.23	9.65	5.65	9.56	7.62	0.26	63.03

Table S2. Fitting result of FT-EXAFS curves shown in **Figure 2c and 2d**.

	Bond type	N	R(Å)	σ^2 ($\times 10^{-3}$Å)
Au_{SA}-MnFeCoNiCu LDH	Au-O	4.316	1.8268	2.2
	Ni-O	5.311	1.9973	1.9
MnFeCoNiCu LDH	Ni-O	5.997	1.9809	2.0

N, coordination number; R, bonding distance; σ^2, Debye-Waller factor.

Error bars that characterize the structural parameters obtained by EXAFS spectroscopy were estimated as N ± 21%; R ± 1%; σ^2 ± 21%.

Table S3. EDS results of 20 Au_{SA}- HE LDHs.

	Cr (at%)	Mn (at%)	Fe (at%)	Co (at%)	Ni (at%)	Cu (at%)	Zn (at%)	O (at%)	Au (at%)
Au_{SA}-CrMnFeCoNi LDH	3.65	4.68	5.91	3.68	5.03	-	-	76.84	0.21
Au_{SA}-CrMnFeCoCu LDH	3.64	4.37	4.38	3.88	-	6.81	-	76.66	0.26
Au_{SA}-CrMnFeCoZn LDH	4.51	3.46	4.43	4.45	-	-	5.33	77.52	0.3

Au_{SA}-CrMnFeNiCu LDH	3.75	4.79	5.86	-	6.14	5.07	-	74.14	0.25
Au_{SA}-CrMnFeNiZn LDH	5.41	4.57	5.21	-	4.18	-	5.58	74.77	0.28
Au_{SA}-CrMnFeCuZn LDH	4.31	4.47	5.91	-	-	5.74	4.91	74.37	0.29
Au_{SA}-CrMnCoNiCu LDH	3.32	3.64	--	4.79	5.41	4.63	-	78.00	0.21
Au_{SA}-CrMnCoNiZn LDH	4.83	6.73	-	5.26	5.84	-	4.74	72.38	0.22
Au_{SA}-CrMnCoCuZn LDH	4.72	4.26	-	4.42	-	4.75	4.45	77.20	0.20
Au_{SA}-CrMnNiCuZn LDH	4.31	4.32	-	-	5.39	4.36	5.56	75.81	0.25
Au_{SA}-CrFeCoNiCu LDH	5.36	-	4.38	5.63	5.83	4.92	-	73.64	0.24
Au_{SA}-CrFeCoNiZn LDH	4.18	-	4.80	4.78	5.50	-	5.55	74.95	0.24

Au_{SA}-CrFeCoCuZn LDH	4.35	-	4.51	5.00	-	4.78	5.83	75.26	0.27
Au_{SA}-CrFeNiCuZn LDH	5.38	-	4.94	-	5.35	4.75	4.59	74.78	0.21
Au_{SA}-CrCoNiCuZn LDH	5.18	-	-	5.82	4.94	5.86	5.97	72.00	0.23
Au_{SA}-MnFeCoNiZn LDH	-	3.94	4.13	3.84	4.14	-	4.38	79.32	0.25
Au_{SA}-MnFeCoCuZn LDH	-	4.73	4.76	3.43	-	3.49	3.70	79.63	0.26
Au_{SA}-MnFeNiCuZn LDH	-	5.67	4.30	-	5.03	5.24	5.18	74.30	0.28
Au_{SA}-MnCoNiCuZn LDH	-	5.42	-	4.53	5.53	4.46	5.79	74.02	0.25
Au_{SA}-FeCoNiCuZn LDH	-	-	4.74	5.63	4.45	5.94	4.91	74.04	0.29

Table S4. The loading amounts of Au_{SA}-MnFeCoNiCu LDH and MnFeCoNiCu LDH.

Sample	Au_{SA}-MnFeCoNiCu LDH	MnFeCoNiCu LDH
Loading of catalyst (mg/cm^2)	0.99	1.02

Table S5. Stability comparison of Au_{SA}-MnFeCoNiCuOOH and other LDHs/oxyhydroxides OER catalysts.

Sample	Current density (mA cm^{-2})	Time (h)	Decay	Ref.
**Au_{SA}-MnFeCoNiCu LDH**	**100**	**700**	**6.4%**	**This work**
FeOOH/NiFe-LDH	100	700	14.1%	1
CoCuFeMoOOH	10/20/50	72	almost no decay	2
EA-FCCN	400	20	7%	3
HEH	10	25	negligible loss	4
HF-HEA-a	10	11	-	5
AN-CuNiFe	10	12	-	6
HEAN@NPC/CC-450	10	24	-	7
CrZnFeCoNi HEHs	70	12	no obvious decay	8
Fe-Cr-Co-Ni-Cu HE-LDHsAr-20	10	16	-	9
NiFe-LDH/Ti_3C_2Tx/NF	50	15	-	10
NiFe LDH	30	24	without any noticeable loss	11
NiFe LDH/V–Co_4N@NF	10/20/50/100	25	-	12

NiO@NiFeLDH/NF-US	50/100	50	-	[13]
NH_3-Fe^{2+} Ni^{2+} -Fe25Ni75	100	24	-	[14]
Ni_3FeW LDH/NF	100	50	-	[15]

Table S6. EDS results of Ru_{SA}-MnFeCoNiCu LDH, Ag_{SA}-MnFeCoNiCu LDH and Pt_{SA}-MnFeCoNiCu LDH.

	Mn (at%)	Fe (at%)	Co (at%)	Ni (at%)	Cu (at%)	Ru (at%)	Ag (at%)	Pt (at%)	O (at%)
Ru_{SA}-MnFeCoNiCu LDH	3.23	6.54	6.42	8.71	7.38	0.30	-	-	67.42
Ag_{SA}-MnFeCoNiCu LDH	3.96	7.21	6.55	8.56	7.93	-	0.31	-	65.48
Pt_{SA}-MnFeCoNiCu LDH	3.58	6.99	7.29	8.14	7.67	-	-	0.28	66.05

Table S7. Calculated LHB values of MnFeCoNiCuOOH and Au_{SA}-MnFeCoNiCuOOH.

	Mn	Fe	Co	Ni	Cu
MnFeCoNiCuOOH	-2.718	-2.699	-2.652	-2.532	-2.475
Au_{SA}-MnFeCoNiCuOOH	-2.779	-2.885	-2.859	-2.908	-2.832

Table 8. Calculated ΔU values of MnFeCoNiCuOOH and Au_{SA}-MnFeCoNiCuOOH.

	Mn	Fe	Co	Ni	Cu
MnFeCoNiCuOOH	3.652	3.429	3.801	3.812	3.650
Au_{SA}-MnFeCoNiCuOOH	3.824	3.865	3.846	4.089	4.013

Table S9. Calculated Gibbs free energy (ΔG) values (U=1.23 V) in AEM on the Mn, Fe, Co, Ni, Cu sites of MnFeCoNiCuOOH and Au_{SA}-MnFeCoNiCuOOH.

	site	ΔG_{*OH} (eV)	ΔG_{*O} (eV)	ΔG_{*OOH} (eV)
MnFeCoNiCuOOH	Mn	-1.11	-1.21	0.71
	Fe	-0.99	-1.32	0.36
	Co	-0.68	-1.32	0.43
	Ni	-0.21	-1.15	0.29
	Cu	-0.87	-1.15	0.49
Au_{SA}-MnFeCoNiCuOOH	Mn	-0.11	-1.18	0.50
	Fe	-0.82	-1.09	0.32
	Co	-0.66	-1.38	0.31
	Ni	-0.65	-1.22	0.08
	Cu	-0.55	-1.17	0.27

Table S10. Calculated Gibbs free energy (ΔG) values in LOM of MnFeCoNiCuOOH with only O vacancy, MnFeCoNiCuOOH with only Au atom, MnFeCoNiCuOOH and Au_{SA}-MnFeCoNiCuOOH.

	ΔG_{*O} (eV)	ΔG_{*OOH} (eV)	ΔG_{Vo+O2} (eV)
MnFeCoNiCuOOH with only O vacancy	0.95	1.75	-0.21

MnFeCoNiCuOOH with only Au atom	0.87	1.56	-1.23
MnFeCoNiCuOOH	0.99	1.75	-0.65
Au_{SA}-MnFeCoNiCuOOH	0.81	1.52	0.38

Supplementary References

1. Liang, Y.*, et al.* Ultrafast Fenton-like reaction route to FeOOH/NiFe-LDH heterojunction electrode for efficient oxygen evolution reaction. *J. Mater. Chem. A* **9,** 21785-21791 (2021).
2. Zhang, L., Cai, W., Bao, N. Top-Level Design Strategy to Construct an Advanced High-Entropy Co–Cu–Fe–Mo (Oxy)Hydroxide Electrocatalyst for the Oxygen Evolution Reaction. *Adv. Mater.* **33,** 2100745 (2021).
3. Zhang, N.*, et al.* Lattice oxygen activation enabled by high-valence metal sites for enhanced water oxidation. *Nat. Commun.* **11,** 4066 (2020).
4. Zhang, T.*, et al.* Boosting the oxygen evolution electrocatalysis of high-entropy hydroxides by high-valence nickel species regulation. *ChemComm* **58,** 7682-7685 (2022).
5. Ma, P.*, et al.* Hydroxylated high-entropy alloy as highly efficient catalyst for electrochemical oxygen evolution reaction. *Sci. China Mater.* **63,** 2613-2619 (2020).
6. Cai, Z.*, et al.* Amorphous Nanocages of Cu-Ni-Fe Hydr(oxy)oxide Prepared by Photocorrosion For Highly Efficient Oxygen Evolution. *Angew. Chem. Int. Ed.* **58,** 4189-4194 (2019).
7. Huang, K.*, et al.* Exploring the impact of atomic lattice deformation on oxygen

evolution reactions based on a sub-5 nm pure face-centred cubic high-entropy alloy electrocatalyst. *J. Mater. Chem. A* **8,** 11938-11947 (2020).

8. Yu, X., *et al.* 2D High-Entropy Hydrotalcites. *Small* **17,** 2103412 (2021).
9. Gu, K., *et al.* Ultrathin defective high-entropy layered double hydroxides for electrochemical water oxidation. *J. Energy Chem.* **60,** 121-126 (2021).
10. Si, H., *et al.* Hydrophilic NiFe-LDH/Ti_3C_2Tx/NF electrode for assisting efficiently oxygen evolution reaction. *J. Solid State Chem.* **295,** 121943 (2021).
11. Suliman, M., *et al.* Growth of ultrathin nanosheets of nickel iron layered double hydroxide for the oxygen evolution reaction. *Int. J. Hydrog. Energy* **47,** 23498-23507 (2022).
12. Zhang, S., *et al.* Heterointerface enhanced NiFe LDH/V–Co_4N electrocatalysts for the oxygen evolution reaction. *J. Mater. Chem. A* **10,** 21523-21530 (2022).
13. Sirisomboonchai, S., *et al.* Fabrication of NiO Microflake@NiFe-LDH Nanosheet Heterostructure Electrocatalysts for Oxygen Evolution Reaction. *ACS Sustain. Chem. Eng.* **7,** 2327-2334 (2019).
14. Chen, Z., *et al.* Revealing the Formation Mechanism and Optimizing the Synthesis Conditions of Layered Double Hydroxides for the Oxygen Evolution Reaction. *Angew. Chem. Int. Ed.* **62,** e202215728 (2023).
15. Wu, L., *et al.* Facile synthesis of nanoparticle-stacked tungsten-doped nickel iron layered double hydroxide nanosheets for boosting oxygen evolution reaction. *J. Mater. Chem. A* **8,** 8096-8103 (2020).
